# Intralesional bivalent and quadrivalent human papillomavirus vaccines didn’t significantly enhance the response of multiple anogenital warts when co-administered with intralesional Candida antigen immunotherapy. A randomized controlled trial

**DOI:** 10.1007/s00403-023-02698-z

**Published:** 2023-08-12

**Authors:** Manal Fawzy, Eman Nofal, Naglaa Abdelkhalek, Rana Ehab

**Affiliations:** Department of Dermatology, Venereology and Andrology, Faculty of Medicine, Zagazig University, Zagazig, Egypt

**Keywords:** Anogenital warts, Bivalent HPV vaccine, Candida antigen, Immunotherapy, Quadrivalent HPV vaccine, Treatment

## Abstract

Treatment of anogenital warts (AGWs) is challenging. Candida antigen immunotherapy has been proven to be a safe and relatively effective therapeutic modality; nevertheless, some patients may experience a partial or no response. Combining Candida antigen with other immunotherapies has been proposed to improve the cure rate. Immunotherapy with human papillomavirus (HPV) vaccines has been tried with conflicting outcomes. This study aimed to  assess the efficacy and safety of intralesional Candida antigen, either alone or in combination with intralesional bivalent or quadrivalent HPV vaccines, for treating multiple AGWs. Eighty patients with multiple AGWs were included and randomly assigned to four equal groups: group A treated with intralesional Candida antigen only; group B treated with intralesional bivalent HPV vaccine (Cervarix) and Candida; group C treated with intralesional quadrivalent HPV vaccine (Gardasil) and Candida; and group D (control) treated with intralesional saline. Complete clearance of lesions was detected in 40%, 20%, and 60% of patients in Candida monotherapy, Cervarix/Candida, and Gardasil/Candida groups, respectively, whereas 40%, 60%, and 20% of patients in the three groups, respectively, showed partial response. Only 10% of the control group had a partial response. Therapeutic outcomes were significantly better in the three treatment groups compared to the control group, with no statistically significant difference between the Candida monotherapy group and the combination groups, but the response was significantly better in the Gardasil/Candida group than in the Cervarix/Candida group. No statistically significant difference was found between the studied groups regarding the development of side effects. Moreover, no recurrence was detected in any of the groups throughout the 3-month follow-up period.  Based on our results, combining intralesional HPV vaccines with Candida antigen immunotherapy may have no significant benefit for treating multiple AGWs. Candida antigen may be recommended as a relatively effective and inexpensive therapeutic modality. The combination of Gardasil and Candida was also effective but very expensive. The results of the Cervarix/Candida combination were unsatisfactory.  This clinical trial was registered and approved prospectively by the ethical review board at Faculty of Medicine, Zagazig University.

## Introduction

Anogenital warts (AGWs) are benign epithelial proliferations that commonly develop in areas prone to abrasion during sexual intercourse. They are caused by human papillomaviruses (HPVs), which comprise a large group of approximately 120 genotypes that infect the skin and mucosa. The majority of HPV infections are subclinical or asymptomatic. However, high-risk HPV types 16 and 18 are the cause of most cervical cancers, while low-risk types 6 and 11 are often associated with AGWs [1]. Lesions of AGWs are often asymptomatic; however, they may cause psychological stress, which is aggravated by the requirement for lengthy and unpleasant therapy. Relapse after seemingly successful treatment is another challenge [2].

Topical, intralesional, and systemic immunotherapies have been widely used for warts because of their nondestructive effect, good safety profiles, promising outcomes, and low relapse rates [3]. They are often used for the treatment of multiple, recalcitrant, or resistant lesions [4]. Intralesional immunotherapy with Candida antigen has been found to be effective and safe for warts, including AGWs. However, complete response was achieved in only some patients [5–7]. Combining Candida antigen immunotherapy with other treatments, including other immunotherapies, was associated with an enhanced response [8–10].

HPV vaccines are widely used to prevent HPV infections by inducing a long-term serum antibody response. They are based on virus-like particles that self-assemble spontaneously from the major capsid protein (L1). They include the bivalent vaccine (Cervarix) that protect against types 16 and 18, the quadrivalent vaccine (Gardasil), that is directed against types 6, 11, 16, and 18, and the nonavalent vaccine (Gardasil 9), against types 6, 11, 16, 18, 31, 33, 45, 52, and 58 [11]. A possible role for these vaccines as a treatment for AGWs has been proposed by several case reports but has yet to be proven in clinical trials [12–14].

Herein, we aimed to assess the efficacy and safety of intralesional Candida antigen immunotherapy alone versus its combination with intralesional bivalent or quadrivalent HPV vaccines for treating multiple AGWs.

## Patients and methods

### Study design

This single-blind, randomized controlled trial was conducted at the Outpatient Clinic of the Dermatology, Venereology, and Andrology Department at Zagazig University Hospitals. The study protocol was reviewed and approved by the Institutional Review Board at Faculty of Medicine, Zagazig University (ZU-IRB#9234/9-1-2022). All patients signed written informed consent to participate in this study**.**

### Participation

During the study period, 83 patients with AGWs were assessed for their eligibility to be included in the study based on specific inclusion and exclusion criteria. Assessment included detailed history taking (personal history, history of present illness, including previous treatment for warts, history of systemic, other dermatological, or sexually transmitted diseases, and history of warts among sexual partners and other family members), and a thorough dermatological examination (to confirm the clinical diagnosis of AGWs, determine the distribution, number, and size of warts, and exclude other dermatological diseases).

Patients aged 12 years old or more from either sex with clinically diagnosed multiple (≥ 5 warts) genital, perianal, or anogenital warts were included. Patients younger than 12 years old, pregnant or lactating females, those with acute febrile diseases, autoimmune disorders, or immunosuppressive conditions, those with a history of serious systemic or anaphylactic reactions to Candida antigen or HPV vaccines, and those who had received any type of wart therapy in the previous month were all excluded.

Based on these criteria, a total of 80 patients with multiple AGWs were enrolled in the interventional phase of the study.

### Therapeutic intervention

Patients were randomly allocated into either one of the four groups of the study (20 patients each) as follows:

*Group A:* received intralesional injection of 0.2 mL of Candida antigen (Specific Hyposensitization Vaccine Candida albicans 1/1000, Immunology Unit, Ain Shams University, Egypt) into the largest wart at 2-week intervals until complete resolution, or for a maximum of 3 sessions.

*Group B*: received intralesional Candida antigen (0.2 mL) alternating with intralesional bivalent HPV vaccine (Cervarix^®^, GlaxoSmithKline, UK) (0.2 mL) into the largest wart at 1-week intervals until complete resolution, or for a maximum of 5 sessions (3 sessions of Candida and 2 sessions of Cervarix).

*Group C*: received intralesional Candida antigen (0.2 mL) alternating with intralesional quadrivalent HPV vaccine (Gardasil^®^, Merck & Co., USA) (0.2 mL) into the largest wart at 1-week intervals until complete resolution, or for a maximum of 5 sessions (3 sessions of Candida and 2 sessions of Gardasil).

*Group D* (the control group): received intralesional injection of 0.2 mL of normal saline into the largest wart at 2-week intervals until complete resolution, or for a maximum of 3 sessions.

### Assessment of therapeutic efficacy

Photographs of lesions were taken at baseline, before each visit, and at the end of therapy. The clinical response was evaluated by two independent dermatologists who were blinded to the treatment assignment. The response was classified as follows: complete response (no visible warts and return of the normal skin markings), partial response (> 50% reduction in wart size and number), and no response (< 50% reduction in wart size and number) [15].

### Assessment of dermatology life quality index (DLQI)

The DLQI questionnaire was performed before and after treatment. It consists of ten questions that aim to measure how much a skin condition affects a patient's daily life. Its score ranges from 0 to 30 [16].

### Safety and follow-up

Any immediate or delayed adverse effects were documented. All patients with complete or partial response were followed up for another 3 months to detect any relapse, while those with no response were referred for other therapeutic options.

### Statistical analysis

Data were collected, tabulated, and statistically analyzed using IBM SPSS Statistics for Windows (Version 23.0. Armonk, NY: IBM Corp.2015). Kruskal–Wallis test, Chi-square test, and Wilcoxon signed rank test were used for data analysis. *P* values less than 0.05 were considered statistically significant.

The percentage of improvement was calculated using the following formula:

$$\% {\text{ of improvement }} = \, \left( {\left[ {{\text{before value}} - {\text{after value}}} \right]/{\text{before value}}} \right) \times {1}00$$

## Results

**Fig. 1:**
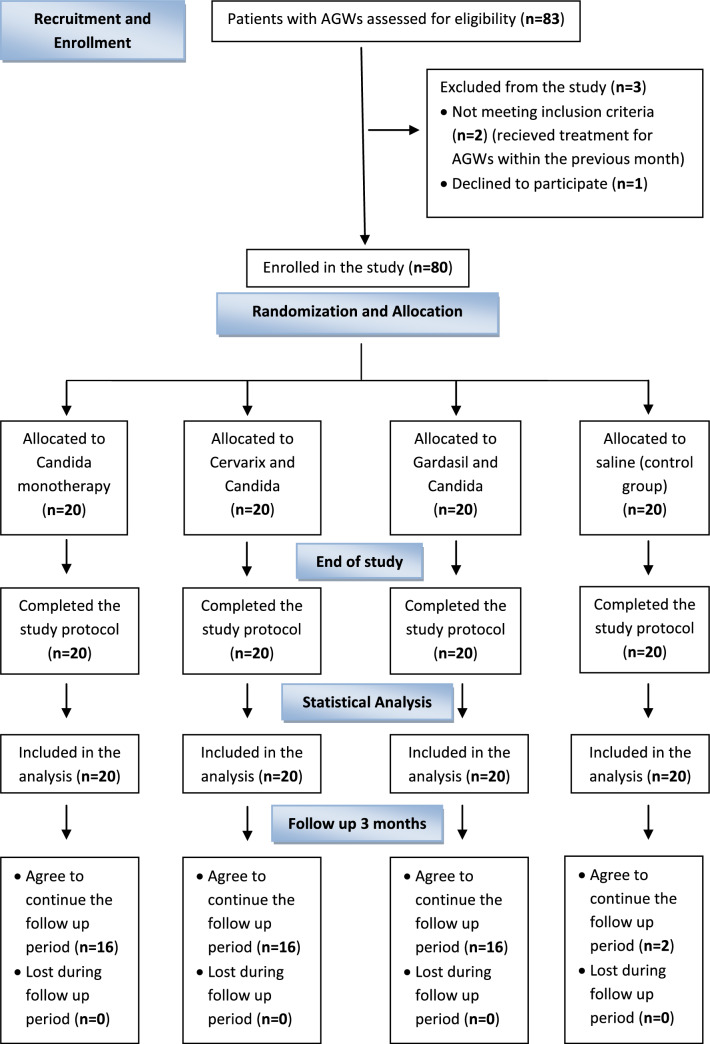
Patients’ flow chart illustrating the sequence of the study according to CONSORT guidelines for reporting randomized controlled trials. *AGWs, Anogenital warts*

Patients’ flow chart is illustrated in (Fig. 1). A total of 80 patients with multiple AGWs were included. They were 62 females (77.5%) and 18 males (22.5%). Their ages ranged from 20 to 66 years, with a mean of 36.2 ± 11.31. The disease duration ranged from 1 to 24 months, with a mean of 8.45 ± 6.57. The size of AGWs ranged from 2 to 20 mm with a mean of 7.3 ± 6.27, while their number ranged from 6 to 35 with a mean of 18.6 ± 15.27. Four patients (5%) were diabetic, two patients (2.5%) had a history of partner affection, and 20 patients (25%) had received cryotherapy previously. The studied groups showed no statistically significant differences regarding the demographic data and baseline disease characteristics (*P* > 0.05) (Table 1).

**Table 1:** Demographic data, history and baseline disease characteristics of the studied patients

Variables	Candida monotherapy group (n = 20)	Cervarix/Candida group (n = 20)	Gardasil/Candida group (n = 20)	Control group (n = 20)	Test	P value
Age (in years)						
Mean ± SD	42.2 ± 13	41.8 ± 11.9	32.6 ± 12.8	39.4 ± 12.1	4.6^a^	0.21
Median (range)	40 (32–66)	40 (25–60)	28 (20–61)	34 (30–65)		
Disease duration (in months)						
Mean ± SD	9.8 ± 8.1	11 ± 8	5.2 ± 3.9	7.8 ± 3.9	3.88^a^	0.26
Median (range)	5 (3–24)	12 (2–24)	4 (1–12)	7 (3–12)		
Size of warts (mm)						
Mean ± SD	8 ± 5.9	4.6 ± 2.95	6.6 ± 5.2	9 ± 7.3	7.3^a^	0.062
Median (range)	4 (3–15)	3 (2–10)	3 (2–15)	4 (3–20)		
Number of warts						
Mean ± SD	18 ± 6.96	20.8 ± 8.6	15.8 ± 10.1	15 ± 4.6	7.4^a^	0.06
Median (range)	15 (10–30)	15 (10–35)	10 (6–35)	15 (10–20)		

	N (%)	N (%)	N (%)	N (%)		
Sex						
Females	12 (60.0)	16 (80.0)	16 (80.0)	18 (90.0)	5.4^b^	0.142
Males	8 (40.0)	4 (20.0)	4 (20.0)	2 ( 10.0)		
Comorbidity (diabetes mellitus)						
Yes	2 (10.0)	0 (0.0)	0 (0.0)	2 (10.0)	4.2^b^	0.24
No	18 (90.0)	20 (100)	20 (100)	18 (90.0)		
Infected partner						
Yes	0 (0.0)	2 (10.0)	0 (0.0)	0 (0.0)	6.2^b^	0.1
No	20 (100)	18 (90.0)	20 (100)	20 (100)		
Previous treatment						
Cryotherapy	4 (20.0)	8 (40.0)	4 (20.0)	4 (20.0)	3.2^b^	0.36
No	16 (80.0)	12 (60.0)	16 (80.0)	16 (80.0)		

P value > 0.05 was considered statistically non-significant

^a^Kruskal-Wallis test

^b^Chi-square test

### Evaluation of the primary outcome (therapeutic efficacy)

**Fig. 2:**
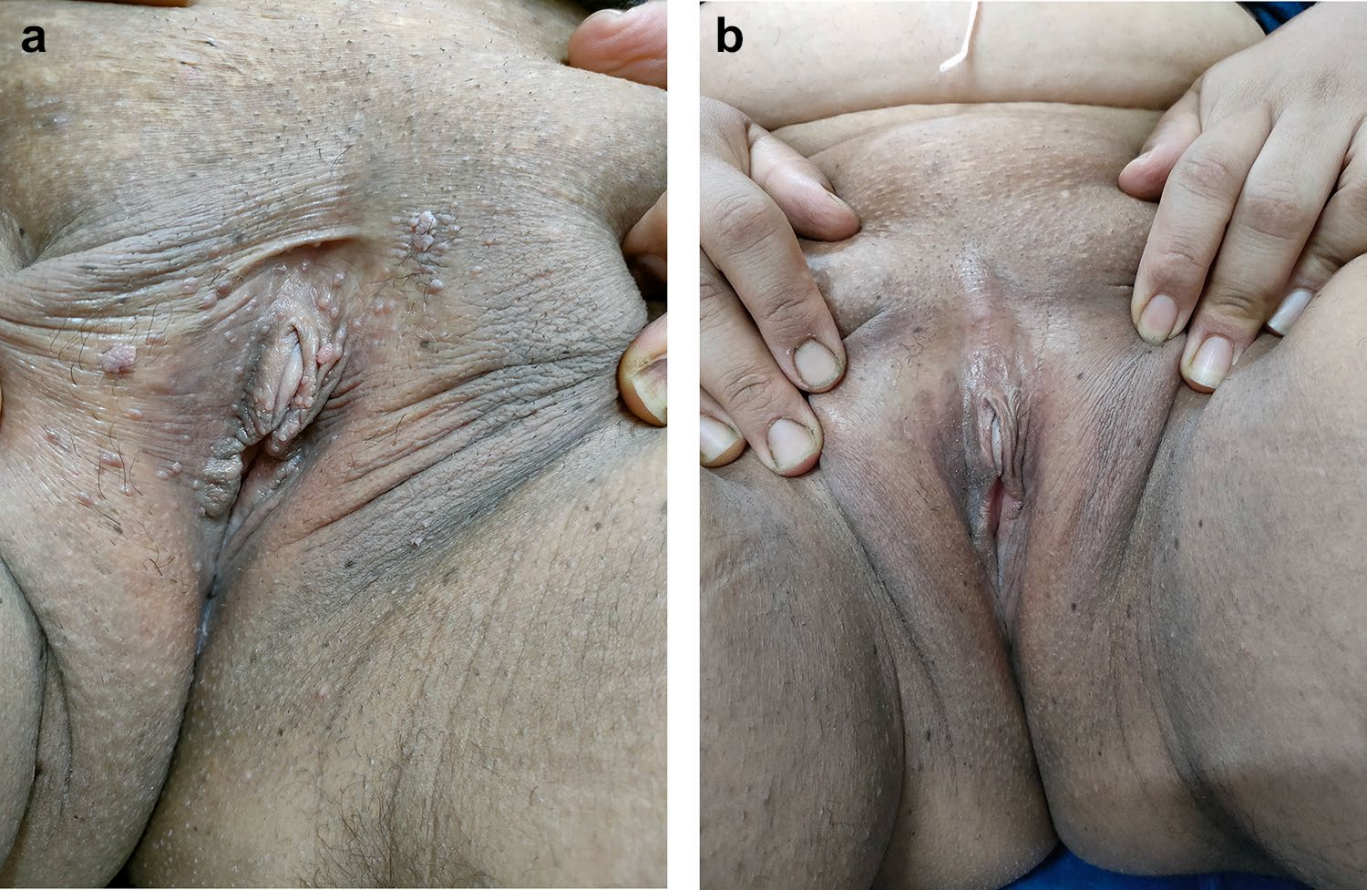
**a** Genital warts before treatment with intralesional Candida antigen immunotherapy **b** Complete clearance of lesions after two sessions (at 4 weeks)

**Fig. 3:**
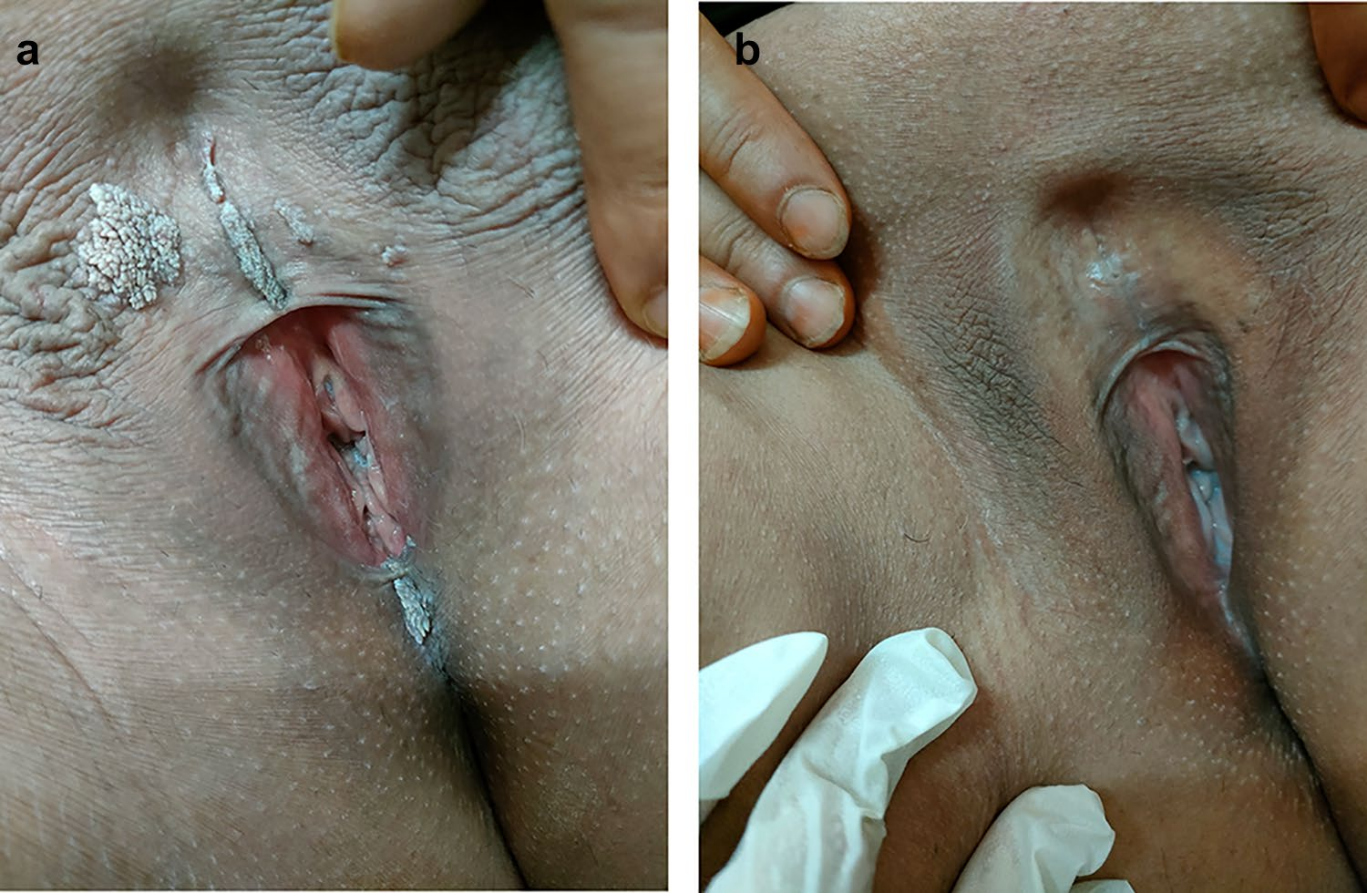
**a** Genital warts before treatment with alternating intralesional injections of Candida antigen and the quadrivalent human papillomavirus vaccine (Gardasil) **b** Complete clearance of lesions after five sessions (at 5 weeks)

**Fig. 4:**
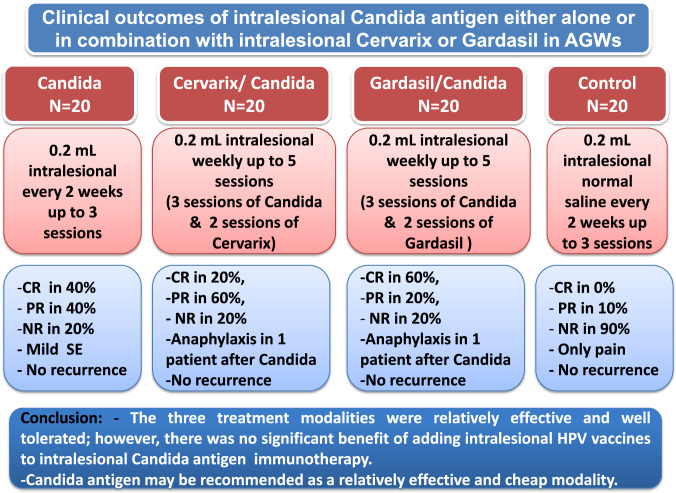
Clinical outcomes of intralesional Candida antigen either alone or in combination with intralesional bivalent (Cervarix) or quadrivalent (Gardasil) human papillomavirus (HPV) vaccines in the treatment of anogenital warts (AGWs). *CR* complete response, *NR* no response,* PR* partial response, *SE* side effects

Complete response was observed in 40%, 20%, and 60% of patients in the Candida monotherapy, Cervarix/Candida, and Gardasil/Candida groups, respectively, while none of the patients in the control group showed complete response. Meanwhile, partial response was noted in 40%, 60%, and 20% of patients in Candida monotherapy, Cervarix/Candida, and Gardasil/Candida groups, respectively. Only 2 patients (10%) in the control group showed partial response. The clinical response was significantly better in the three treatment groups compared to the control group (*P* < 0.001), with no statistically significant difference between the three treatment modalities (*P* > 0.05), except for a significantly better response in the Gardasil/Candida group compared to the Cervarix/Candida group (*P* = 0.018) (Table 2, Figs. 2, 3, 4).

**Table 2:** Degrees of clinical response in the studied patients with anogenital warts

Variable	Candida monotherapy group (A) (n = 20)	Cervarix/Candida group (B) (n = 20)	Gardasil/Candida group (C) (n = 20)	Control group (D) (n = 20)
	N (%)	N (%)	N (%)	N (%)
Degree of response				
Complete response	8 (40.0%)	4 (20.0%)	12 (60.0%)	0 (0.0%)
Partial response	8 (40.0%)	12 (60.0%)	4 (20.0%)	2 (10.0%)
No response	4 (20.0%)	4 (20.0%)	4 (20.0%)	18 (90.0%)
P value^a^	< 0.001*	< 0.001*	< 0.001*	Reference group

P1^b^ (comparing groups A, B, C) = 0.092

P2^b^ (comparing groups A & B) = 0.344

P3^b^ (comparing groups A & C) = 0.344

P4^b^ (comparing groups B & C) = 0.018*

*P value < 0.05 was considered statistically non-significant

^a^Comparing each treatment group with the control group (reference group) using Chi square test

^b^using Chi square test

The wart size and number were significantly reduced in all groups except the control group. Although there were no statistically significant differences between the four groups regarding wart size and number before treatment, both parameters were significantly lower in the treatment groups than in the control group after treatment, with a significantly higher percentage of change in the treatment groups compared to the control group (Table 3).

**Table 3:** Wart characteristics (size and number) and the Dermatology Life Quality Index (DLQI) in the studied patients with anogenital warts

Group	Variable	Before treatment	After treatment	% of improvement	W^a^	P value
Wart size (mm)						
Group A	Mean ± SD	8 ± 5.9	4.6 ± 5.8	62 ± 38.1	3.1	0.002*
	Median (range)	4 (3–15)	2 (0–15)	60 (0–100)		
Group B	Mean ± SD	4.6 ± 2.95	2.8 ± 3.8	55.3 ± 33.2	3.6	< 0.001*
	Median (range)	3 (2–10)	1 (0–10)	60 (0–100)		
Group C	Mean ± SD	6.6 ± 5.2	1.1 ± 2.3	73.3 ± 39.9	3.5	< 0.001*
	Median (range)	3 (2–15)	0 (0–10)	100 (0–100)		
Group D	Mean ± SD	9 ± 7.3	8.1 ± 6.6	6.5 ± 16.9	1.7	0.089
	Median (range)	4 (3–20)	4 (2–20)	0 (0–60)		
KW^b^		7.3	28.5	28.7		
P value		0.062	< 0.001*	< 0.001*		
Number of warts						
Group A	Mean ± SD	18 ± 6.96	8.95 ± 11.3	60.3 ± 38.5	3.5	< 0.001*
	Median (range)	15 (10–30)	7 (0–30)	53.3 (0–100)		
Group B	Mean ± SD	20.8 ± 8.6	8.3 ± 8.9	59.99 ± 25.5	3.8	< 0.001*
	Median (range)	15 (10–35)	6 (0–35)	60 (0–100)		
Group C	Mean ± SD	15.8 ± 10.1	6.7 ± 11.6	75.2 ± 34.2	3.9	< 0.001*
	Median (range)	10 (6–35)	0 (0–35)	100 (0–100)		
Group D	Mean ± SD	15 ± 4.6	13.95 ± 5.3	7.8 ± 18.1	1.7	0.098
	Median (range)	15 (10–20)	15 (4–20)	0 (0–60)		
KW^b^		7.4	17.1	32.9		
P value		0.06	< 0.001*	< 0.001*		
DLQI						
Group A	Mean ± SD	9.6 ± 2.4	4.2 ± 4.5	58.3 ± 397	3.5	< 0.001*
	Median (range)	10 (6–12)	4 (0–12)	58.3 (0–100)		
Group B	Mean ± SD	9.9 ± 5.3	5 ± 3.6	52.9 ± 32.7	3.5	< 0.001*
	Median (range)	9 (5–29)	5 (0–13)	57.1 (0–100)		
Group C	Mean ± SD	7.6 ± 3.1	1.6 ± 2.1	65 ± 44.7	3.51	< 0.001*
	Median (range)	7 (4–12)	0 (0–5)	100 (0–100)		
Group D	Mean ± SD	9.6 ± 1.5	8.95 ± 1.8	5.2 ± 13.5	1.7	0.1098
	Median (range)	10 (8–12)	8 (5–12)	0 (0–50)		
KW^b^		6.6	33.9	27.3		
P value		0.087	0.001*	< 0.001*		

Group A (Candida monotherapy), Group B (Cervarix/Candida combination), Group C (Gardasil/Candida combination), Group D (Control)

*P value < 0.05 was considered statistically significant

^a^W: Wilcoxon signed rank test (for comparison within each group)

^b^KW: Kruskal Wallis test (for comparison between groups)

### Evaluation of DLQI

The DLQI was significantly reduced in the treatment groups only. There was no statistically significant difference between the studied groups regarding the DLQI before treatment (*P* = 0.087), while there was a significant difference between them after treatment in favor of the treatment groups (*P* < 0.001), with a significantly higher percentage of change in the treatment groups compared to the control group (*P* < 0.001) (Table 3).

### Adverse effects

All patients had tolerable pain during the injection. Anaphylaxis developed in 2 patients (one in Cervarix/Candida group and one in Gardasil/Candida group) 15 min after the last Candida antigen injection. Both patients improved after anti-shock measures (intramuscular adrenaline 1/1000, intravenous hydrocortisone sodium succinate 100 mg, and intravenous pheniramine 45.5 mg). Itching was reported in 17 patients in the treatment groups only. There was no statistically significant difference between the studied groups regarding the development of side effects (*p* > 0.05) (Table 4).

**Table 4:** Adverse effects of the studied treatment modalities among patients with anogenital warts

Adverse effects	Candida monotherapy Group (n = 20)	Cervarix/Candida Group (n = 20)	Gardasil/Candida Group (n = 20)	Control Group (n = 20)	χ^2^	P
	N (%)	N (%)	N (%)	N (%)		
Pain	20 (100%)	20 (100%)	20 (100%)	20 (100%)	–	–
Itching	5 (25%)	6 (30%)	6 (30%)	0 (0.0)	7.4	0.06
Anaphylaxis	0 (0.0)	1 (5%)	1 (5%)	0 (0.0)	2.05	0.56

P value > 0.05 was considered statistically non-significant

*χ*^*2*^ Chi square test

### Recurrence

No recurrence was detected among patients with complete or partial response during the 3-month follow-up period.

## Discussion

The current treatment options for AGWs have variable efficacy, and optimal treatment continues to be required [11]. Among these options is intralesional Candida antigen immunotherapy, which has been reported to be safe and effective in treating warts, including AGWs [5–7].

In the current study, 40% of patients in the Candida monotherapy group had complete clearance of AGWs. Marei et al. [10] reported a similar rate of complete clearance of recalcitrant warts in 40% of 20 patients (including 12 patients with genital warts) following intralesional Candida antigen. On the contrary, our results were slightly lower than those reported by Elmaadawy et al. [6], who observed a complete clearance rate of 50% in 20 patients with AGWs after intralesional Candida antigen. This slight variation could be attributed to the longer interval in their study (every 3 weeks) than in our study (every 2 weeks), which could impact the immunological response.

Also, our results were lower than those of Tawfik et al. [7], who reported complete clearance of AGWs in 62.5% of 40 patients following intralesional Candida antigen. This disparity could be due to the smaller sample size and lower number of sessions (3 sessions) in our study compared to their study (4 sessions). Furthermore, they conducted a presensitization test and excluded patients with a negative reaction because they were predicted to be non-responders, while we didn’t conduct this step, and therefore this may explain the better response in their study.

The precise mechanism of intralesional Candida antigen immunotherapy is not fully understood. This modality is mostly associated with stimulation of T-helper (Th)-1 cytokine production, such as interferon (IFN)-γ and interleukin (IL)-2, which stimulate natural killer cells and cytotoxic T cells to eradicate HPV infection [17]. Also, Candida cell wall components may bind to toll-like receptors (TLRs), primarily TLR-2 and TLR-4, inducing the production of tumor necrosis factor (TNF)-α and type I IFNs that eliminate viral infections [18]. Furthermore, it has the potential to activate the complement system, resulting in chemotaxis of various inflammatory cells and direct destruction of virally infected cells via a membrane attack complex [19].

To enhance the response rate of intralesional Candida antigen immunotherapy, its combination with other therapeutic modalities, including various immunotherapies, has been investigated [8–10]. Combining more than one immunotherapy has been proposed to simultaneously target several immune pathways, resulting in greater results [3]. On the other hand, several trials have demonstrated varying efficacy of HPV vaccines either as monotherapy or in combination with other treatments for warts [10, 20–25]. Hence, the current study aimed to assess the efficacy of adding intralesional HPV vaccines (Cervarix and Gardasil) to intralesional Candida antigen immunotherapy in treating multiple AGWs.

In the present study, the clinical response was significantly better in the three treatment groups compared to the control group. Surprisingly, the Cervarix/Candida group had a lower complete response rate (20%) than the Candida monotherapy group (40%), while the Gardasil/Candida group had a higher complete response rate (60%) than the Candida monotherapy group. However, no statistically significant difference was detected between the Candida monotherapy group and combination groups, but the response was significantly better in the Gardasil/Candida group compared to the Cervarix/Candida group. This could imply that Gardasil is superior to Cervarix as an immunostimulant; yet, it didn't significantly improve the clinical response to Candida immunotherapy. In consistency with our results, Gilson et al. [20] reported no advantage from adding the 3-dose intramuscular regimen of Gardasil to either imiquimod or podophyllotoxin cream for treating AGWs.

On the contrary, other studies have reported the efficacy of HPV vaccines, either as monotherapy or in combination with other therapies, for warts. Nofal et al. [21] reported that intralesional Cervarix yielded complete response in 45.5% of 22 patients with AGWs. This rate was higher than our rate in the Cervarix/Candida group (20%). We were unable to explain this discrepancy, but the use of only two Cervarix sessions (due to alternating injection with Candida antigen) versus 5 sessions in their study, as well as the shorter duration of treatment (4 weeks versus 8 weeks in their study) may have contributed to our lower results.

Furthermore, Marei et al. [10] compared the efficacy of combining 3 doses of intramuscular Cervarix (0.5 ml at 0, 1, 6 months) with Candida antigen (0.2 ml/ session for a maximum of 5 sessions) versus Candida antigen alone in 40 patients with recalcitrant warts (22 of whom had genital warts). The combination group had significantly higher complete response rate (70%) than the monotherapy group (40%) (P = 0.014). Moreover, their Cervarix/Candida group had a substantially higher complete response rate than ours (70% versus 20%). This could be attributed to the difference in the Cervarix administration route. Interestingly, Nofel et al. [22] detected a non-significant superiority of intralesional Cervarix over its intramuscular administration in patients with recalcitrant common warts. However, our results (using the intralesional route) were lower than those of Marei et al. [10] (using the intramuscular route). This discrepancy could be due to other differences, including the longer treatment duration in their study (6 months versus 4 weeks in our study) and the larger amount of Cervarix and Candida antigen in their study, which may have resulted in a much better immune response.

Meanwhile, Choi [23] reported that a 3-dose schedule of intramuscular Gardasil resulted in complete clearance in 60% of patients with genital warts, which was comparable to our rate in the Gardasil/Candida group, demonstrating that the response of this combination didn’t significantly differ from monotherapy. However, the key advantages of the combination in our trial were the shorter treatment duration (4 weeks versus 6 months in their study) and the smaller amount of Gardasil (only one vial per patient versus 3 vials in their study), making the combination more cost effective.

Additionally, Protasov et al. [24] found that the combination of imiquimod 5% cream with Gardasil resulted in complete response in 94.4% of 36 patients with AGWs within one year. Their outcomes were much better than ours. This could be related to differences in the route of Gardasil administration, as well as the longer duration of therapy and follow-up in their study.

Furthermore, Kumar et al. [25] observed that intralesional Gardasil resulted in complete resolution of recalcitrant genital warts in 33.3% of patients at week 6. This rate was lower than our rate in the Gardasil/Candida group (60%). Therefore, intralesional Gardasil/Candida combination could actually be more effective than intralesional Gardasil monotherapy. Unfortunately, our study didn't include an intralesional Gardasil monotherapy group to support this assumption. Therefore, further research is required to evaluate this probability.

Although HPV vaccines are primarily used to prevent HPV infections by inducing the production of antibodies that bind to the virions, their mechanism as immunotherapy for warts has not yet been established. This may be due to their ability to evoke both B-cell and T-cell responses that cross react with other HPV strains, resulting in wart clearance and prevention of their recurrence. They have been shown to induce long-lasting protective immunity by stimulating circulating B memory cells, which release neutralizing antibodies upon subsequent antigenic exposure. Also, they have been shown to up-regulate levels of Th1 cytokines, such as TNF-α and IL-2, as well as pro-inflammatory cytokines, such as IL-1α, IL-1β and IL-6 [26].

In the present study, only 10% of patients in the control group had partial response that may be explained by psychological factors or may reflect the natural course of the disease with possible spontaneous regression [27].

Regarding side effects, anaphylaxis was observed in 2 patients (in the combination groups) upon the last Candida antigen injection and was treated immediately with anti-shock measures. Pain and itching at the injection site were also detected. There was no statistically significant difference between the studied groups. Similar side effects were reported in previous studies [6, 7, 10, 21, 25].

During the 3-month follow-up period, no recurrence was detected among patients with complete or partial response. Similarly, other studies have reported no recurrence in patients who had received Candida antigen [6], HPV vaccines [21, 23, 24], or both [10].

## Limitations

Limitations of the current study include the limited number of patients and the short follow-up duration. Moreover, we didn’t compare HPV vaccine monotherapy with Candida antigen monotherapy or their combinations.

## Conclusion

There was no significant benefit of adding intralesional HPV vaccines to Candida antigen immunotherapy for treating multiple AGWs. Candida antigen may be recommended as a relatively effective and inexpensive modality. The combination of Gardasil and Candida is also effective but highly expensive. Recommendation of Cervarix could not be validated by the current study. Further larger studies are needed to confirm the efficacy of HPV vaccines administered intralesionally or intramuscularly, either alone or in conjunction with other treatments. The immunological response in HPV vaccine recipients should be evaluated to gain more insight into their mechanism of action. Furthermore, the influence of HPV genotypes on the responsiveness of AGWs to various immunotherapies should be explored.

## Data Availability

The data that support the findings of this study are available from the corresponding author upon reasonable request.
